# Why don't patients seek help for chronic post‐surgical pain after knee replacement? A qualitative investigation

**DOI:** 10.1111/hex.13098

**Published:** 2020-07-09

**Authors:** Andrew J. Moore, Rachael Gooberman‐Hill

**Affiliations:** ^1^ Musculoskeletal Research Unit Translational Health Sciences Bristol Medical School Southmead Hospital University of Bristol Bristol UK; ^2^ National Institute for Health Research Bristol Biomedical Research Centre University Hospitals Bristol NHS Foundation Trust and University of Bristol Bristol UK

**Keywords:** arthroplasty, replacement, knee, chronic pain, help‐seeking behaviour, motivations, osteoarthritis, pain, post‐operative, physician–patient relations, qualitative research, United Kingdom

## Abstract

**Background:**

Although many people are satisfied with their outcome after total knee replacement surgery for osteoarthritis, around 20% report chronic post‐surgical pain. People are often disappointed and unsure about whether their pain is normal and what can be done about it. Given the high prevalence of long‐term post‐operative pain after knee replacement, there is potentially a large hidden population with an unaddressed need for care.

**Objective:**

In this study, we focus on understanding why some people choose not to consult health care for chronic post‐surgical pain after knee replacement.

**Methods:**

Semi‐structured interviews were conducted with people who had received total knee replacement, at either of two National Health Service hospitals in the United Kingdom, and who had chronic post‐surgical pain (n = 34, age 55‐93 years). Data were audio‐recorded, transcribed and analysed thematically.

**Results:**

We found an overall sense of futility amongst participants who believed that nothing further could be done for their on‐going pain. People's perception of their pain was often discordant with that of surgeons and physicians. Other factors that contributed to decisions not to seek help included low expectations about effectiveness and the risks involved in further treatment, treatment burden, participants' prioritization of other health conditions and views about candidacy. Many accepted their on‐going pain.

**Conclusion:**

Our study indicates why some people with chronic pain after knee replacement do not seek further health care. Understanding patients' beliefs and expectations about chronic post‐surgical pain can inform approaches that might enable people to seek help in the future.

## 1 INTRODUCTION

Osteoarthritis is a long‐term condition that is associated with joint pain, swelling and stiffness that can lead to loss of function.^1^ Ten per cent of UK adults consult primary care for osteoarthritis every year.^2^ UK guidelines for the management of osteoarthritis recommend non‐surgical treatments in the first instance (information, education, exercise, physiotherapy, weight‐loss, medication). Although these may relieve pain and improve function in early stage osteoarthritis, many people will eventually undergo joint replacement.^3^ In the UK during 2017, 112 836 primary knee replacements were performed of which around 96% were for osteoarthritis.^4^ For many, knee replacement can provide substantial improvements in pain and function with the largest gains primarily within the first 3 months with some improvement after that.^5^, ^6^, ^7^ However, best quality studies show that 20% of people report chronic post‐surgical pain after total knee replacement.^8^ The International Association for the Study of Pain defines chronic post‐surgical pain as pain developing or increasing in intensity after a surgical procedure, persisting beyond the healing process, that is at least 3 months after surgery, and in the area of preceding surgery.^9^ People with chronic post‐surgical pain are often disappointed with their post‐operative outcome.^10^, ^11^ Some may feel abandoned by health care, particularly if surgeons downplay the significance of pain.^12^ Patients may struggle to make sense of on‐going pain and blame themselves for poor outcomes.^13^ Such pain remains under‐recognized despite evidence that it affects between 10% and 50% of patients who undergo surgery.^14^ Wylde et al suggest that part of the problem of chronic post‐surgical pain is that it can occur after many different surgical procedures, and therefore, no single medical specialty has ‘ownership’ of the problem.^15^

Treatment options for the management of chronic pain include referral to physiotherapy, surgery, medication and psychological pain management approaches which can all be accessed through the UK's primary care services provided through the publicly funded National Health Service (NHS). However, for patients who present with chronic post‐surgical pain after knee replacement, there are no clear referral and treatment pathways and health‐care professionals often see no clear way to help patients.^16^, ^17^ A large proportion of older adults with chronic musculoskeletal pain do not use health services for their pain and those living with multiple health conditions are more likely to believe that pain is an inevitable part of ageing, or that little can be done.^18^, ^19^, ^20^, ^21^ Given the high prevalence of chronic post‐surgical pain after knee replacement, there is potentially a large hidden population who do not seek care for their pain.^8^

Understanding the complex nature of why some people decide to seek care or not is important for ensuring timely diagnosis and treatment. Previous research with people with joint pain shows that help seeking (either new or follow‐up consultations) comprises a dynamic interplay between individual meaning making, knowledge gained from social networks, relationships with health‐care professionals, and socio‐political and moral discourses about consultation behaviour.^22^ Theoretical models of health and illness behaviour such as the Model of Pathways to Treatment can provide a useful way of understanding help‐seeking behaviour.^23^

**Figure 1:**
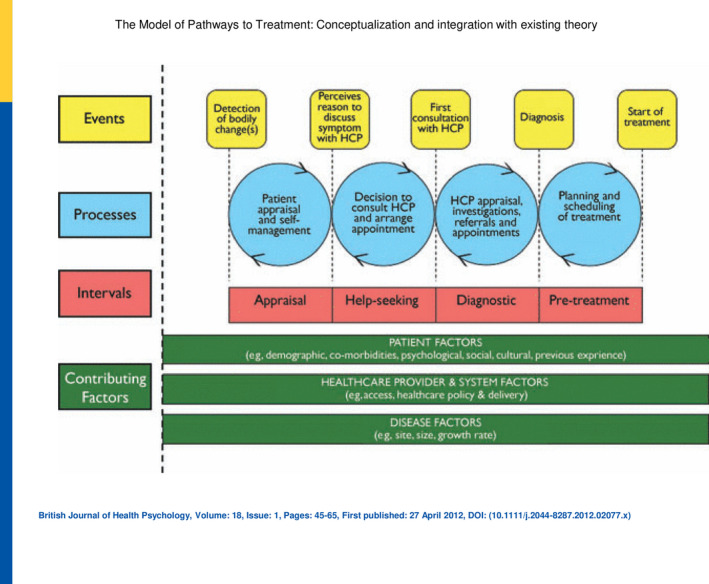
The model of pathways to treatment

The Model of Pathways to Treatment encompasses existing psychological theory to explain the events and processes that occur during help seeking, including decisions not to seek help.^23^ The model describes ‘backwards and forwards’ bi‐directional movements between a series of events and processes (Figure 1). This begins with the patient detecting a bodily change during the appraisal process which leads to determining a reason to seek help; a decision to consult then leads to the first consultation; the clinicians' appraisal leads to a diagnosis; and finally, the planning of treatment leads to the start of treatment.

The model is underpinned by elements from social cognitive theory.^24^ These include self‐efficacy—the confidence people have in their ability to bring about desired outcomes,^25^ and outcome expectancies—the perceived consequences of an individual's actions, which include incentives and disincentives to help seeking.^24^, ^26^ The model has been widely used to understand help‐seeking behaviour in cancer populations but previously with people with chronic pain after knee replacement.^27^, ^28^, ^29^ Our aim in this study was to understand why some people with chronic post‐surgical pain after total knee replacement choose not to seek help. We draw on concepts from the Model of Pathways to Treatment in the discussion to explain these findings.

## 2 METHODS

### 2.1 Identification of participants

The study was conducted in the United Kingdom with patients who had received total knee replacement at either of two high‐volume National Health Service hospitals in Central and South‐West England. We sampled purposively, choosing a target of 40 to ensure we achieved a diverse range of participants, including patients who were between 12 months and 5 years post–knee replacement from across both the Central and South‐West England regions of the UK. We aimed to recruit equal numbers across this time frame, by posting out invitations in phases across each 12‐month period. We chose this time frame because people can continue to experience some improvement in pain and function up to 12 months post‐operatively^7^ and we wanted to understand non‐use of services for chronic post‐surgical pain in the longer term. A clinical team member identified potential participants from patient lists.

Potential participants received an information pack and screening questionnaire about pain and health‐care use (see Appendix S1). The design of the questionnaire and definition for the threshold of inclusion was determined in discussion with pain clinicians, research methodologists and a patient and public involvement (PPI) group. The questionnaire included elements of the Level of Expressed Need Scale^30^ and the complete Oxford Knee Score,^31^ which is widely used in national programmes to assess outcomes relating to pain and function. Both scores are validated. Patients were eligible for inclusion if they answered ‘yes’ to the question: ‘Are you currently troubled by pain in your replaced knee, either all the time or on and off, which has lasted for more than 3 months?’, and scored between 0 and 14 on the seven Oxford Knee Score pain questions, and described seeing GPs or other health‐care professionals in relation to their pain as ‘rare’ or ‘never’ in the previous 12 months. The screening questionnaire was discussed with patient representatives who thought that these terms were appropriate.

Eligible patients interested in participating were contacted by a member of the research team [AJM] to arrange a face‐to‐face interview. All interviews took place in participants' homes. The study was conducted in compliance with the Declaration of Helsinki, and ethical approval was granted by the West Midlands—Solihull Research Ethics Committee (Reference number 15/WM/0469). All participants provided written informed consent.

### 2.2 Data collection

Interviews were conducted by an experienced qualitative methodologist who was previously unknown to the participants [AJM]. Interviews lasted between 32 minutes and 105 minutes (mean average 57 minutes). Data collection took place between May 2016 and August 2018. A semi‐structured topic guide was used to guide discussion, and to enable the researcher and participant to explore and reflect on different areas. Topics included experience of chronic pain after knee replacement, characteristics of pain, comorbidities, self‐management and use of formal and informal health services. The guide was designed in collaboration with members of the study's patient and public involvement group (Appendix S2).

### 2.3 Data analysis

**Figure 2:**
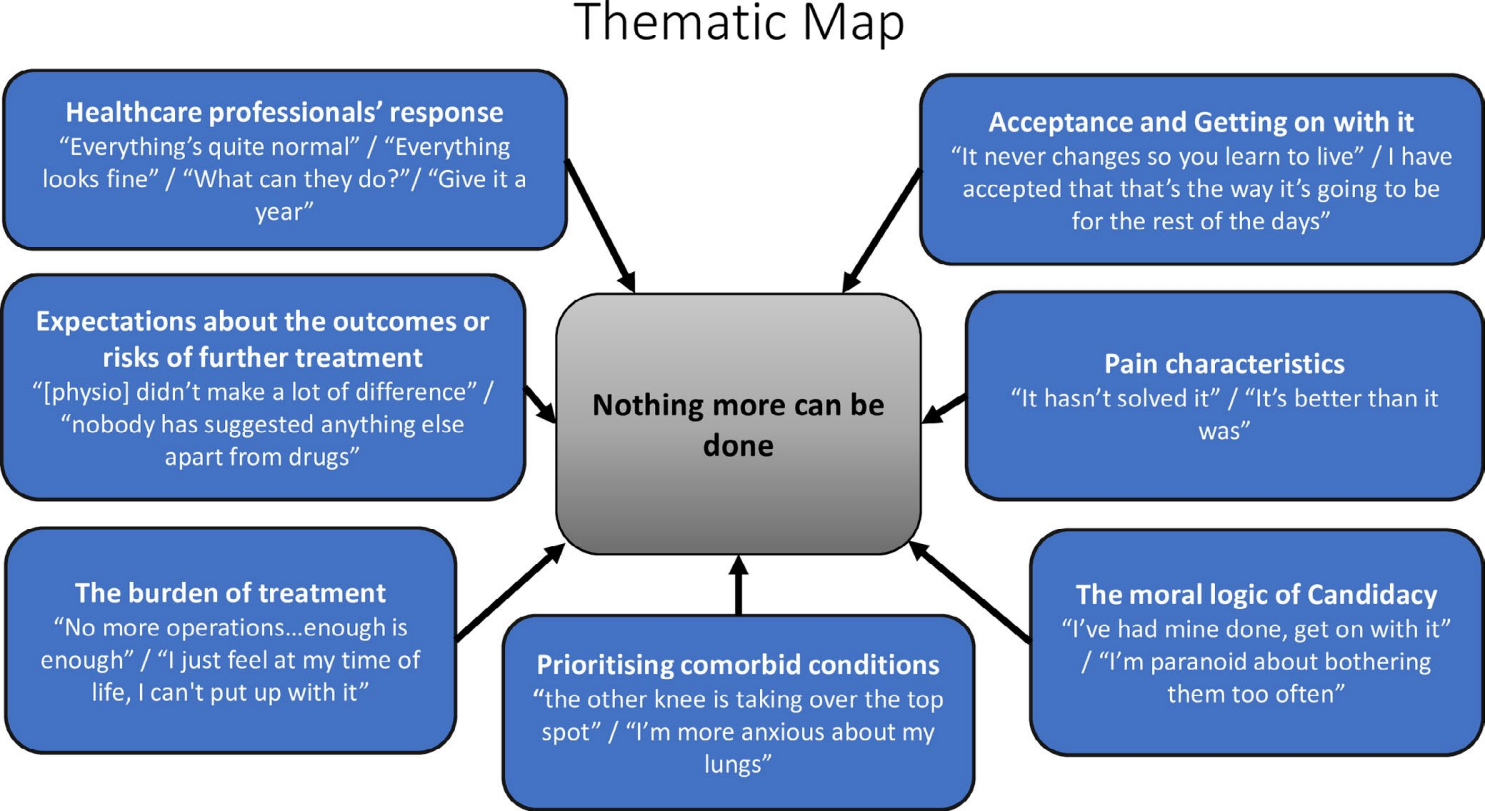
Thematic map

Inductive thematic analysis^32^ was undertaken by AJM with additional input from RGH and FM, all experienced qualitative methodologists with backgrounds in musculoskeletal and pain research [AJM, RGH, and FM] health sociology [AJM] and anthropology [RGH]. Interviews were audio‐recorded, transcribed, anonymized and uploaded to QSR NVivo 11 data management software. Data relevant to the research question were coded across the data set and two other team members [RGH and FM] independently coded four transcripts. Developing codes were discussed and refined and then applied across the data set and reviewed. Those that shared unifying features or patterns were grouped into themes (Appendix S3). At this point, a thematic map was constructed to illustrate the themes and the presence of an overarching ‘core theme’ (See Figure 2).

Our approach was inductive and the codes were developed from and grounded within the data.^33^

## 3 RESULTS

Thirty‐four people took part (18 women), all of whom had received a total knee replacement between 14 and 68 months before interview (Table 1). Average age was 74 years, ranging from 55 to 93 years.

**Table 1:** Participant characteristics

Participant characteristics	Number (n)
Age group, y	
56‐64	6
65‐74	12
75‐84	12
>85	4
Gender	
Male	16
Female	18
Time post–knee replacement at interview, years	
1‐2	7
2‐3	11
3‐4	13
>4	3

A single core theme encapsulated the overall sentiment: ‘nothing more can be done’ (see Figure 2). Seven related subthemes explain why participants had rarely or never sought health‐care for their pain. We include illustrative quotes relating to these subthemes, and all names used are pseudonyms.

### 3.1 Nothing more can be done

Overall, participants described a sense of futility and a belief that nothing further could be done for their pain. This belief was informed by a combination of experiences and expectations about health care and whether further care was appropriate or possible. These are contained in the seven inter‐related subthemes: the response of health‐care professionals; expectations about outcomes or risks involved in further treatment; the burden of treatment; prioritizing other health conditions; the moral question of candidacy; characteristics of pain; and acceptance.

### 3.2 **Health‐care professionals**'**responses to chronic post‐surgical pain**

Many participants' beliefs about chronic post‐surgical pain were influenced by the response of surgeons and general practitioners (GPs). During standard follow‐up appointments, participants often suggested their experience of recovery was discordant with the surgeon's view of success. If surgeons expressed satisfaction with radiographs and could see no technical or mechanical reason for on‐going pain (eg misalignment of the prosthesis), people were then left with a sense of uncertainty about the cause of their pain and whether it was part of normal recovery:

I have mentioned this, when I've been at the [hospital] for check‐up and he said well we've seen the x‐ray of the knee replacement and he said everything's quite normal.(Dave)

This was often the case if surgeon's suggested that the pain was normal or to be expected:

I did go back to see Mr [consultant], which you do, I told him I was still having some pain, ‘Well, that's normal’(Phoebe)

Hilda described how she was repeatedly told that her recovery would take time: ‘They just kept saying ‘give it a year and it should improve’ and it never did’—she was still experiencing pain fifty‐one months post‐surgery at the time of interview.

Initial consultations with GPs were also seen as unhelpful, as patients described receiving offers of pain medication which they did not want.

Well nobody seemed to want to help me do anything about it. The GP didn't particularly want to help. He just wanted to give me stronger painkillers(Eric)

Others did not consult their GP because they expected that nothing further could be done or offered, having already had a joint replacement.

I don't know what else she would offer.(Claire)

### 3.3 Expectations about the outcomes or risks of further treatment

Some participants decided not to engage further with health care because they felt that doing so was risky. Some thought that they might be referred for further surgery, which they wished to avoid for fear it could worsen their outcomes, and in one case even result in amputation.

I don't want to tempt fate. I've got a lot of problems with the heart and what have you and things can happen(Rory)

The last time I saw the registrar at the hospital [about 15 months after the surgery] he said to me then, he said, ‘You've got two options’. He said, ‘We can either take that joint out, have a look, see what's happening, put a new joint in’. He said, ‘That might improve the situation, it might not improve it. It might make it worse’. He said, ‘The other option is just leave it as it is until it gets to the stage where it's totally unbearable and we've got to replace it’.(Eric)

My old doctor said to me in the end, when I told him, I said about, you said, you've got to live with it. There's nothing anybody else could do for you. I remember [physiotherapist], saying to me, ‘The only way we can cure that is to take your leg off’(Oscar)

Having chronic post‐surgical pain also affected people's decisions about whether to have surgery on other painful joints and to risk worsening their situation.

When [surgeon] said, ‘No,’ there was nothing that could be done, I said, ‘In that case, I'm not having the other one done ‘cause I'm not going to have both knees hurting […] it's constant and it's really painful.(Harriet)

Some also assumed that further consultation with a GP, or a specialist pain clinic, would lead to further unwanted medication or interventions, such as physiotherapy, that people felt were ineffective.

I saw the doctor probably a year or 18 months ago and they just give you, you know, tablets […] and the last thing I want to do is take – keep taking tablets.(Benjamin)

I'd have thought the physio was going to be the answer and although I enjoyed the six weeks I did it, I don't think any more of that would help.(Nora)

### 3.4 The burden of treatment

As well as concerns about the outcomes of further treatment, some participants wanted to avoid the personal disruption caused by surgery and recovery, including incapacitation, being away from home or enduring months of waiting before an operation. Others felt they were too old to go through the physical exertion of further treatment, especially when they were coping with other health conditions. Oscar had undergone thirteen operations altogether, for his knee and other problems and felt that ‘enough is enough’.

You've got to wait probably two or three months now, haven't you? And then I've got to go through that; so, you know, five or six weeks which I'm more or less tied to this flat. So, I think to myself, ‘Well, I'll put up with the pain’. It might not sound logic to you.(Benjamin)

I just feel at my time of life, I can't put up with it [more surgery], do you know?(Rory)

### 3.5 Prioritizing comorbid conditions

Some participants were also living with conditions that took priority over their post‐surgical pain. Benjamin explained that his other knee had become more painful and that he was also living with a terminal lung condition, which was his main focus.

I'm more anxious about my lungs when I'm short of breath. I'm anxious, then I think to myself, ‘This is it' […] That, that, to me is my – at the moment, for me, it's the lungs that are priority.(Benjamin)

Similarly, Peter's heart problem caused breathlessness and limited his ability to mobilize.

As I said to you, it takes me all the time to get to the kitchen, breathless. Now that is my biggest concern, breathlessness, not me knee. My knee is purely in the background.(Peter)

One patient felt that although her knee was painful, she could manage it, and the pain in her foot concerned her more. Clint also suggested that the pain from his replaced knee was ‘bearable’ compared to that of his other knee which was ‘taking over the top spot’.

### 3.6 The moral logic of candidacy

Importantly, there was also a moral dimension to people's decision about whether they were suitable candidates to consult health care. Some participants felt they had already received a fair share of health care, and were mindful of the financial cost of treatment, and believed that if they were to seek further help then this might delay care for others who were in greater need.

Well what are they going to do about it? ‘Well I'll refer you to the hospital’ [adopts mocking tone]. And then you wait then, so like a year, and you're taking up a valuable appointment for somebody else who's in far more pain and discomfort than I am, I've had mine done, get on with it, get over it, you know, do you know what I mean? I'm extremely grateful to the NHS, I've had thousands of pounds spent on me you know with my hip and my two knees, and I am much better obviously than I was before; the fact that this is still painful just gives me something else to moan about really.(Gwen)

Gwen also questioned the worth of consulting as although her knee was still painful, she felt it was ‘something else to moan about’. Even though Elsa had been asked by the surgeon to contact him if there were further problems, she also felt that others were possibly in more need of treatment.

I can't phone her up and say ‘Oh, I got a pain’. [interviewer: why not?] Well, he's seeing to people, that really needs to have it done. And, I mean, I've had mine done.(Elsa)

Participants also spoke about how they did not want to complain or criticize because they thought that doctors were under pressure. Claire was also mindful that she chose to have the operation, which suggests that some patients feel that the responsibility for managing pain after their operation, rested with them rather than health care.

You've had the surgery, so … they've done what you've asked, haven't they? […] I'm paranoid about bothering them too often. […] also, I know this sounds funny but, if you go back and complain you almost feel as if you're criticising their work.(Claire)

### 3.7 Pain characteristics

All participants reported a high level of pain severity during screening before they were invited to interview. During interviews, they were asked about the characteristics of their pain and whether it had changed over time. Some reported that although they still experienced episodes of intense pain, it was considerably better than pre‐operatively, which made it easier to cope with.

I don't like the sharp ones, when I get them that is awful, you know, but I do tolerate it with medication, because, like I said it's erm, it's a breeze to what it was, yeah.(Brenda)

I'm not in as much pain now as I was before I had the operation. I have to admit that. But I'm in sufficient pain to make it a nuisance and to make it difficult for the rest of my life.(Hilda)

Some participants continued taking medication to manage their pain and focussed on the improvement in function, or compared themselves with others they thought were ‘worse off’ than themselves.

I'm extremely grateful for the amount that I can do, so I suppose the pain, if you were to sort of weigh it up, I suppose the improvement is much better than the pain, does that make sense?(Gwen)

For some these improvements, although marginal, made on‐going pain more acceptable, and were a disincentive to seek further help.

### 3.8 Acceptance and getting on with it

Many participants spoke of ‘just getting on’ with their lives, describing an acceptance of their situation because they felt that there was no other choice.

The pain's there. So what? You've got other things to do, get on with it. And that's just the way it is.(Bella)

Well, there's no alternative. If you could tell me an alternative to not accepting the fact.(Oscar)

Others rationalized that because they were older and less active, pursuing solutions to their pain was not worth the effort.

Obviously at [my age] you ain't got 40 years to live have you […] I don't see there's very much else left that I can do’—Donald.

With different levels of acceptance, all participants were trying to self‐manage their on‐going pain.

### 3.9 Bringing the themes together in the context of the model of pathways to treatment

In the context of the Model of Pathways to Treatment (Figure 1) a person's detection of a bodily change (which might include a lack of improvement in pain), and appraisal of whether they have a reason to consult for on‐going pain is undermined if clinicians suggest that their pain is a normal part of recovery. Participants' concerns that further treatment might be painful, ineffective or risky, or that the burden of treatment may further disrupt their lives is represented by physical outcomes expectancies in the model. Participants' moral concerns about criticizing or wasting clinicians' time or burdening the health‐care system appear as social outcome expectancies, while concerns about being a ‘good citizen’ by not delaying care for others and by accepting one's situation and self‐managing are represented by self‐evaluative expectancies. Participants reported that being older, prioritizing comorbid conditions and pain severity acted as disincentives to help seeking, and these appear as patient factors in the model. The analysis indicates that people with pain do not move on to seek help and become trapped in a futile loop in which they think there is a good reason to seek help but that there are a range of considerations that prevent them from doing so.

## 4 DISCUSSION

**Figure 3:**
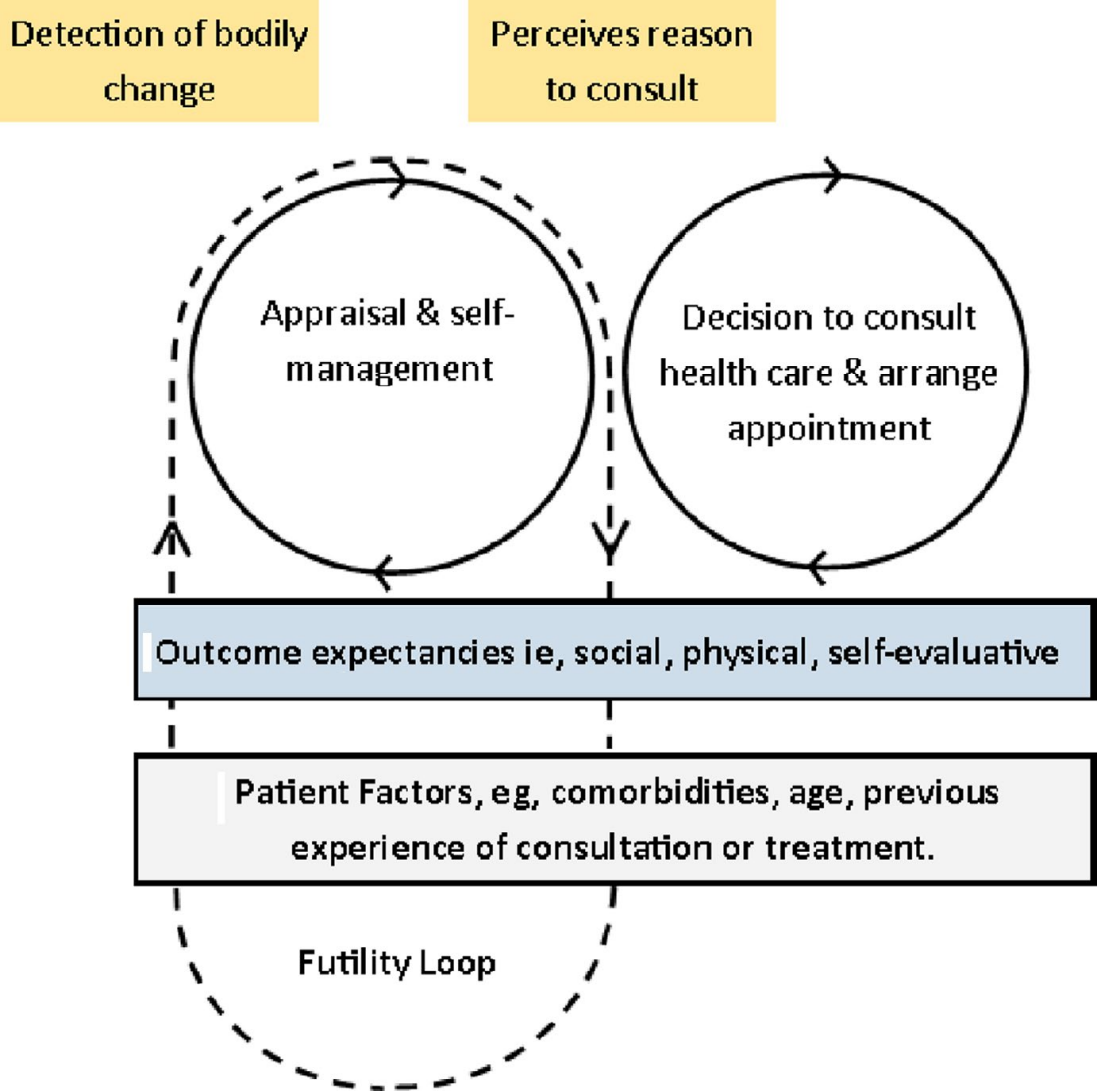
The futility loop

The overarching theme that ‘nothing more can be done’ reflects a common experience. Based on their initial experience of follow‐up appointments or seeking support for chronic post‐surgical pain many participants had stopped seeking help, for multiple reasons leading to a sense of futility. This experience may be conceptualized as a ‘futility loop’ (Figure 3), whereby patients become trapped between the appraisal and help‐seeking interval, in which they think that that there could be a reason to consult for pain but that nothing further can be done.

Participants often felt the responses of health‐care professionals were discordant with their own experience of on‐going pain. Where there was no technical or mechanical reason for on‐going pain, surgeons' assertions that the operation was a success and that pain was normal left people uncertain about whether they could seek further care. Importantly, seeking further care was also felt to be risky: more surgery may worsen the pain, or the likely outcome was not thought to be worth the effort, and for some people other health conditions took priority. Most participants in our study also wanted to avoid medication either because of unwanted side‐effects or their view that medicines were ineffective.

Participants' narratives also suggest that their decisions to seek health care are influenced by moral discourses surrounding the responsible and appropriate use of a publicly funded health‐care system, the National Health Service (NHS). The use of moral discourses are a known consideration in health‐care choices, for instance Townsend and colleagues^34^ describe moral dilemmas in people's accounts of living with chronic illness. Previous studies have highlighted that patients are highly sensitive to demands on the NHS and are anxious not to be seen as timewasters or unworthy candidates for treatment ^35^, ^36^ Some participants in this study expressed this in terms of having had an adequate share of services, where having a knee replacement has constituted an appropriate allocation of health care. They were also concerned that their further use of health care might delay care for others they felt had more need. Participants also appeared to view surgery as the final ‘fix’ or ‘solution’ for osteoarthritis and were uncomfortable with ‘bothering’ health‐care professionals with on‐going pain. Other studies have suggested that people do not consult health‐care professionals for fear of ‘bothering’ them^37^ or time‐wasting^38^ and that people may compare the seriousness of their condition with others whom they think are in greater need.^22^

Toye reported that people with chronic musculoskeletal pain felt that health‐care professionals disbelieved their pain leading to them feeling delegitimized.^39^ However, patients in our study report that while clinicians acknowledged their pain, it was often normalized. We also found that some patients avoided further consultations for fear that it might lead to more surgery and worse outcomes. Previous research highlights that health‐care professionals in the NHS report an absence of clear access points into care for people with post‐surgical pain.^16^ This finding perhaps resonates with participants' experiences in this study and may explain their view that nothing further could be done.

Some participants felt that help seeking involved a personal burden and suggested they were too old to expend further efforts to redress the balance of health and illness. This is supported by previous research which suggests that as people get older they are less inclined to seek help for chronic pain.^40^ The sense of personal burden and the context of ageing also resonate with literature that describes the ‘hard work’ of managing osteoarthritis before surgery and shows how people may reduce self‐management efforts as they age.^41^, ^42^, ^43^ Our work extends these findings, showing them to be as relevant even after surgical intervention for osteoarthritis.

Many participants suggested they were ‘getting on with it’ because they felt there was no alternative, other than to accept their situation and self‐manage. Jeffrey et al ^12^ found that patients' level of acceptance of chronic pain after knee replacement varied according to the severity of pain and how much improvement or deterioration in their life circumstances they had experienced after knee replacement. For some in our study, constant pain had dramatically changed their physical and social well‐being. For others, their pain was less than pre‐surgical pain levels and some improvement appeared to make this more acceptable. Many people felt they had no other option than to just accept pain and get on with things. Lorig and Holman^44^ suggest ‘*One cannot not manage. … it is impossible not to manage one's health. The only question is how one manages’*. Patients are also used to managing pre‐surgical knee pain due to osteoarthritis in this way,^42^, ^45^ so when surgery fails to alleviate pain, then reverting to similar self‐management strategies makes sense. Patients also prioritized some comorbidities, such as pulmonary and cardiac conditions, which were felt to limit function or represent more of a future risk than chronic post‐surgical pain, similar to findings from other studies.^46^, ^47^

We used the Model of Pathways to Treatment,^23^ to provide an explanatory framework for how people seek health care. The appraisal and help‐seeking intervals are particularly relevant to participants in our study as all participants had cycled through the pathway as they sought treatment for osteoarthritis pain, ultimately leading to their knee replacement. Following this, some cycled through once or twice as they sought help for pain, but most had either never sought, or had stopped seeking help and had become stuck between the appraisal and help‐seeking intervals in what we call the futility loop (Figure 3). According to the model, during the appraisal interval an individual may become aware of bodily sensations (pain, stiffness) or visual information (swelling, rash), which might indicate something is abnormal. However, participants in our study were often told at their early follow‐up appointments that their pain was normal, to be expected, and would improve over time. As people progressed through recovery, and pain remained troublesome, many saw a good reason to discuss their symptoms with a health‐care professional but never moved on to the help‐seeking stage, because of their expectations about the likely outcome. This is explained in the model by inclusion of elements of Bandura's Social Cognitive Theory,^48^, ^49^ which describe outcome expectancies, the anticipatory factors that influence behaviour within the help‐seeking interval. This resonates with why patients in this study decided not to seek care. Bandura suggests that unless patients believe their actions will produce desired effects, for example relief from post‐surgical pain, they will be less inclined to seek help because of these outcome expectancies. Bandura specified three forms of outcome expectations^48^, ^49^:

*Physical outcome expectancies* could disincentivize help seeking if people believe that help seeking could result in further pain, ineffective or unpleasant treatment, or disruption to their lives, such as the patients in our study who did not want further surgery or medication.

*Social outcome expectancies* involve social sanctioning and reactions to help‐seeking behaviour. For example, some participants in our study did not seek help partially for fear they would be viewed as time‐wasting or ungrateful, or placing further burden on the NHS. When participants asked about their on‐going pain, their concerns were delegitimized, by radiographic evidence, or health‐care professionals' assertions that symptoms were normal, thus taking away their reason to seek help in the future.

*Self‐evaluative outcome expectancies* describe how people self‐evaluate and self‐sanction according to their own personal standards and moral values. For example, individuals want to be seen as ‘good citizens’ and participants felt that nothing further can be done, and made a decision to self‐manage, and not to overburden the NHS, this helps to maintain a positive self‐identity.

Barriers to health‐care use have been the focus of this study. The barriers described provide some explanation for why people do not proceed through the appraisal interval and on to help seeking. Scott et al^23^ acknowledge that other events, beyond the stages that they already identify, may occur throughout the pathway and beyond. However, they caution that identifying further events would not be useful as they would be too acute, ill‐defined or varied to measure across different contexts. On the contrary, we found that after completion of treatment (knee surgery), patients may well return to the appraisal and self‐management process, but then fail to move on to help‐seeking because of a sense of futility and an overall belief that ‘nothing more can be done’. We describe this as becoming stuck in a ‘futility loop’.

### 4.1 Strengths and limitations

A qualitative approach has enabled us to explore the complex experiences and views of people living with chronic post‐surgical pain after knee replacement. Our broad purposive sample of thirty‐four participants provided sufficient data to achieve data saturation, the point at which the collection of new data becomes unnecessary,^50^ and we are confident that our sample was sufficiently diverse and that our use of an inductive approach has provided thematic areas of greatest relevance and salience to participants. Although we did not collect information on reasons for non‐participation, the diverse sample and achievement of saturation means that we are confident that the findings are transferable to other people within the same population in the UK, however, we recognize that health systems vary between contexts and countries. Using the Model of the Pathways to Treatment to explain the findings facilitated the interpretation of barriers to help‐seeking behaviour and has enabled us to add more broadly to the literature through the concept of the futility loop. Our study did not include the accounts of clinicians as we were interested in understanding people's reasons for non‐use of health care, and so needed to hear patients' detailed accounts and their interpretation of events and their meaning. However, previous research reports that clinicians also feel that care pathways for patients with chronic post‐surgical pain after knee replacement are unclear and they often struggled to help patients.^16^ Future research might focus on clinicians' interpretation of patients' help‐seeking behaviour for post‐surgical pain. Although complications after surgery can be associated with on‐going pain, we did not request access to medical records because our study focused on experience rather than clinical reports of complications. Two participants reported that they developed infection after surgery, which cleared after antibiotic treatment. They did not associate the infection with their on‐going pain which developed later. One patient also reported nerve damage due to the surgery and described this as implicated in their pain.

## 5 CONCLUSION

Our research explains why some people with chronic pain after knee replacement do not seek further health care. Understanding these reasons may help health care professionals to better prepare patients for knee replacement surgery. Our findings suggest that health care professionals' responses to patients' disclosure of pain can powerfully affect patients' beliefs about whether they have a legitimate reason to consult for on‐going pain. We recommend that all patients should be informed about the possibility that there may be some persistent pain post‐operatively, and that while some pain in the early post‐operative period is normal, patients should re‐consult if pain persists. It is also important that at follow‐up appointments clinicians acknowledge the presence of pain and enable patients to feel able to come forward for care should the pain persist.

Our work also identifies that some patients think there is a risk in further consultation which might lead to unwanted treatment and worse outcomes. We suggest that health care professionals ensure that patients have information about all local health care services and treatments to help them achieve better pain management, with information on how to access these. Patients who experience a moral conflict concerning further consultation for their replaced joint and over‐burdening the NHS should also be helped to understand that asking for support for their pain is appropriate and may be helpful. There is a need for further research to identify other kinds of support and treatment that might prevent people with pain from becoming stuck in the futility loop. The identification of the futility loop has particular value for people living with long‐term pain and who have undergone a number of, often major, interventions over time.

## CONFLICT OF INTEREST

The authors declare no conflicts of interest.

## Supporting information

Appendix S1

Appendix S2

Appendix S3

## Data Availability

The data that support the findings of this study are available on request from the corresponding author. The data are not publicly available due to privacy or ethical restrictions.
