# Supplementary material for: Why don't patients seek help for chronic post‐surgical pain after knee replacement? A qualitative investigation
Source: Health Expect. 2020 Jul 9;23(5):1202–12. doi: 10.1111/hex.13098 (PMC7696127; doi:10.1111/hex.13098)
Supplement: Supplementary file 1 — Appendix S1 [file HEX-23-1202-s001.pdf]

SuppInfo1_Screening Questionnaire

Screening Questionnaire for Pain and health care use after total knee replacement

These questions ask about long-term pain after knee replacement and what help you have received for it. Your answers will help us to find people with different experiences of pain and healthcare use to take part in a research project. **If you have read the enclosed information booklet and have completed the consent form and are willing to fill in the questions below**, then please answer the following questions and return this questionnaire with the consent form and reply slip in the FREEPOST envelope. Whatever you decide about participation in the study your decision will not affect the care you receive. Thank you for your time.

Please **tick** Yes or No

Are you currently troubled by pain in your replaced knee, either all the time or on and off, which has lasted for more than three months?	YES		NO	

If you answered 'NO' to the question above, thank you for your time. Please return this questionnaire to us in the FREEPOST envelope. There is no need for you to answer the next questions, but returning the form to us will mean that we do not trouble you with a reminder letter or about the rest of the research. Thank you.

If you answered 'YES' to the question above, please could you complete the next two pages of questions and also return this form to us in the FREEPOST envelope. Thank you.

The following questions ask about problems during the past 4 weeks with your knee that has been replaced. Please tick (✓) one box only for each question.

1) How would you describe the pain you usually have from your knee?

None	Very mild	Mild	Moderate	Severe

2) Have you had any trouble with washing and drying yourself (all over) because of your knee?

No trouble at all	Very little trouble	Moderate trouble	Extreme difficulty	Impossible to do

Continued on next page….

3) Have you had any trouble getting in and out of a car or using public transport because of your knee? (whichever you tend to use)

No trouble at all	Very little trouble	Moderate trouble	Extreme difficulty	Impossible to do

4) For how long have you been able to walk before pain from your knee becomes severe? (with or without a stick)

No pain/More than 30 minutes	16 to 30 minutes	5 to 15 minutes	Around the house only	Not at all/pain severe when walking

5) After a meal (sat at a table), how painful has it been for you to stand up from a chair because of your knee?

Not at all painful	Slightly painful	Moderately painful	Very painful	Unbearable

6) Have you been limping when walking, because of your knee?

Rarely/never	Sometimes or just at first	Often, not just at first	Most of the time	All of the time

7) Could you kneel down and get up again afterwards?

Yes, easily	With little difficulty	With moderate difficulty	With extreme difficulty	No, impossible

8) Have you been troubled by pain from your knee in bed at night?

No nights	Only 1 or 2 nights	Some nights	Most nights	Every night

9) How much has pain from your knee interfered with your usual work (including housework)?

Not at all	A little bit	Moderately	Greatly	Totally

Continued on next page….

10) Have you felt that your knee might suddenly 'give way' or let you down?

Rarely/never	Sometimes, or just at first	Often, not just at first	Most of the time	All of the time

11) **Could** you do the household shopping on your own?

Yes, easily	With little difficulty	With moderate difficulty	With extreme difficulty	No, impossible

12) Could you walk down one flight of stairs?

Yes, easily	With little difficulty	With moderate difficulty	With extreme difficulty	No, impossible

The following question is about health care use.

Which of these statements most accurately describes your health care use in the **last 12 months** in relation to your **knee pain**?

	Please tick one
I see my GP and other health care professionals about my knee pain as and when I need to.	
I rarely see my GP **or** other health care professionals about my knee pain.	
I no longer see my GP **or** other health care professionals about my knee pain.	
I have never seen a GP **or** other health care professional about my knee pain.	

Your name (please print) Mr / Mrs / Ms / Miss...

If you would like any further information about these questions, or help filling them in, please telephone [Researcher contact details removed].

For office use only: to be completed by research staff

Site				Study ID			
