# Supplementary material for: Why don't patients seek help for chronic post‐surgical pain after knee replacement? A qualitative investigation
Source: Health Expect. 2020 Jul 9;23(5):1202–12. doi: 10.1111/hex.13098 (PMC7696127; doi:10.1111/hex.13098)
Supplement: Supplementary file 2 — Appendix S2 [file HEX-23-1202-s002.pdf]

SuppInfo2_Topic Guide

Investigating the management of pain after total knee replacement: Patient Topic Guide

Introduction and consent

Discuss how the interview will be recorded, issues of confidentiality, anonymisation and the aim of the research.

Focus of the interviews: We want to explore how people think about the pain they have, and how it has affected their life as well as the support that people have received for their pain in the past and at present.

Socio-demographic data *…these questions are to help me understand a little more about your background so that I can understand how your condition might affect you…*

- Marital status/employment status/dependents/ living situation/other health conditions/needs/ hobbies/?

Pain after Knee Replacement (*detection of bodily changes*)

- Focussing on the knee you had replaced and in which you have long term pain, can you tell me what happened after your knee replacement operation once you had been discharged from hospital?
- When did the pain start?
- How has it changed over time?
- Is it different to the joint pain you had before your replacement? How? *Quality / duration / frequency*

Causes of pain

I'm interested in what other people think about the causes of their pain? Do you mind if I ask you a few questions about that?

- Have you ever thought about what's causing the pain?
- Do you ever try to picture what is happening within the joint to cause that pain? Do you have a picture in your mind of what it is?
- Do you ever *worry* about what might have caused the pain? *Worry about why you have it?*

Seeking Health Care *(reasons for discussing symptoms with HCP)*

- After your knee replacement when did you first start to become aware that your pain was not improving?
- How were you managing your pain at that point?
- Did you talk to others about your pain? *Family / others who received TKR*

- Is there a point at which you started to become concerned or anxious about the pain?
- What did you do in terms of seeking help for your pain? *Did you visit your GP or Consultant?*
- How long was it before you sought help? (days, weeks or months)
- What happened when you sought help?
- What sort of advice did you receive about your pain? - *How did that make you feel (satisfied/unsatisfied)?*
- Did anyone offer a diagnosis? – *What was it?*
- Did you have follow-up appointments with your consultant after your knee replacement? *How often? Did you talk about the pain then?*
- Tell me about the advice and treatment you've received for your pain – *Has it helped? / did it make any difference?*
- What sort of things have your tried to help your pain? (health care or otherwise)
- Do you take any prescription or non-prescription medication for your pain? *If prescription who prescribed them?*
- How do you manage your pain at the moment? *what helps / does not help?*
- Do you manage your pain differently now from when you first had pain after your knee replacement?
- Up to now, which healthcare professionals have you spoken to in regards to your ongoing pain?
- What support do you currently have for your knee pain? *GP / physiotherapy / social support / psychological or counselling support / alternative practitioners (acupuncture, osteopathy etc)*
- Are there other health care professionals you would like to speak to, or someone you wish to speak to again?
- Have you ever been to a specialist pain clinic for your knee pain? *Who referred you / was this helpful*
- Is there anything that health care providers (GPs, hospitals etc) do, which you find unhelpful when seeking help for your pain?
- What has it been like trying to find help and support for your pain? *Is there anything that complicates this?*
- Has there ever been a period during which you felt like you had given up looking for health care support? *How long for? Why?*

Impact of pain

- How has the pain you have now affected your life? (daily activities, relationships, work, leisure, mood)
- How do you feel about the future in regards to your pain?
- If you wanted health care professionals to focus on any part of your care now, what would it be?
- What do you think health care professionals could do to improve care for people who have long-term pain after knee replacement?

About participating in this study

- Is there anything about your participation in this research that you would have liked to have happened differently?

Conclusion

- Is there anything else you would like to add, or anything you wish to talk about that we haven't covered already?
- Would you like us to send you a brief report of the study findings?

Reaffirm consent…thank you for participating…END.
