# Supplementary figures and images for: Why don't patients seek help for chronic post‐surgical pain after knee replacement? A qualitative investigation
Source: Health Expect. 2020 Jul 9;23(5):1202–12. doi: 10.1111/hex.13098 (PMC7696127; doi:10.1111/hex.13098)

## SuppInfo3\_Coding Tree

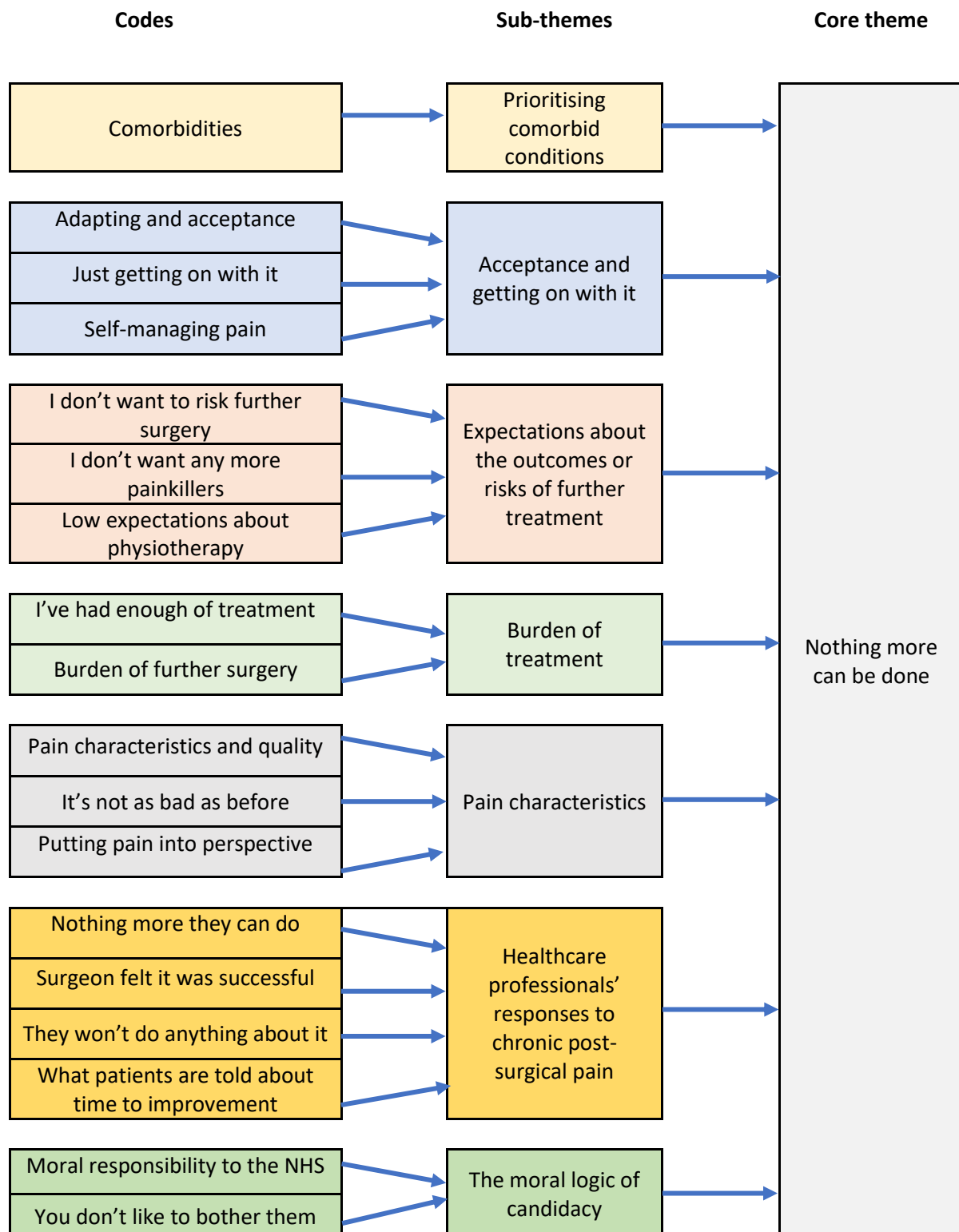

Supplement: Supplementary file 3 — Appendix S3 [file HEX-23-1202-s003.pdf]
